# Anaemia among mother-father-child pairs in India: examining co-existence of triple burden of anaemia in a family

**DOI:** 10.1186/s12889-021-11408-1

**Published:** 2021-07-07

**Authors:** Pradeep Kumar, Shekhar Chauhan, Ratna Patel, Shobhit Srivastava

**Affiliations:** International Institute for Population Sciences, Mumbai, Maharashtra 400088 India

**Keywords:** Anaemia, Triple burden, Family, India, National family health survey

## Abstract

**Background:**

Anaemia is a global health concern and is also a common comorbidity in multiple medical conditions. Very limited research is available examining anaemia among family members in India and across various countries. The present study aimed to examine the co-existence of the triple burden of anaemia among mother-father-child pairs in a family.

**Methods:**

The data utilized was from the National Family Health Survey conducted in 2015–16. The effective sample size for the study was 26,910 couples, along with children aged 6–59 months. The bivariate and binary logistic regression analysis were applied to assess the factors associated with family-level anaemia. In bivariate analysis, a chi-square test was performed to determine the association of socio-demographic factors with anaemic family.

**Results:**

More than half of the mothers (57.5%) and their children (58%), along with 10% of fathers, were found to be anaemic; however, the co-existence of triple burden of anaemia among mother-father-child pairs was 4.7% in the study. The likelihood of family-level anaemia was low when both the parents were educated [OR: 0.69, CI: 0.58–0.81], and it was high when both the parents were employed [OR: 1.40 CI: 1.10–1.80]. Families from the Scheduled Tribe had a 62% higher likelihood to suffer from anaemia [OR: 1.62, CI: 1.33–1.97].

**Conclusions:**

The suggested interventions include early diagnosis, effective management, and treatment of anaemia. Moreover, adequate complementary feeding practices for children shall also be promoted. Parental education on nutrition is also required, and community interventions are needed to improve parental education on nutrition. At last, there is a need for greater policy and program attention to improving nutritional knowledge among mothers so as to tackle the triple burden of anaemia among mother-father-child pairs.

## Background

Anaemia is a global health concern and is also a common comorbidity in multiple medical conditions [1]. Anaemia is a condition that affects all age groups; however, it is more prevalent during the first two years of life, during adolescence and during pregnancy among women [2]. Around one-third (32.9%) of the world’s population was estimated to suffer from anaemia [3]. Anaemia was found to be highest among pre-school children aged 0–5 years (43%), followed by pregnant women and girls aged 15–49 years (38%), non-pregnant women and girls aged 15–49 years (29%), and school-age children aged (25.4%) [3]. Based on a nationally representative cross-sectional study, Didzun et al. (2019) found that the prevalence of anaemia was 23.2%, moderate or severe anaemia was 5.1%, and severe anaemia was 0.5% among Indian men [4]. Another study in EAG states of India noticed that around one-fourth of the men were suffering from anaemia [5]. In the current scenario, the situation has not improved much, and children and women continue to be highly affected by anaemia as compared to men [1].

Anaemia among children is a widely studied topic [6–8]. Various studies have noted specific determinants of anaemia among children. Household wealth and maternal anaemia were associated with anaemia among children [7]. Another study noted that maternal education plays a significant role in controlling anaemia among children [6]. Another study noted maternal education, residence, and wealth index correlate with childhood anaemia [9]. Similarly, various studies have also examined the correlates of anaemia among women [10, 11]. Household wealth index and place of residence were found to be significant determinants of anaemia among women [11]. Men have been mainly ignored in anaemia-focused research and policies [12]. The limited availability of literature on anaemia among men makes this study important in the current context. A study has found that the prevalence of anaemia among men has remained unchanged (23%) in the last 10 years, from 2006 to 2016 [10]. The same study confirmed that higher levels of anaemia among men were found in those residing in rural areas, employed in more labour-intensive activities, and belonging to the lowest wealth quintile [12].

Anaemia among children in India has studied extensively [13]. Similarly, anaemia among women is one of the well-documented topics, and literature is available in abundance, examining factors associated with anaemia among women [14]. Anaemia has always been a topic of immense interest among researchers in India; however, nearly all the research on anaemia is attributed to either children or women [15, 16]. Not only in India but also in other countries too, literature about anaemia is limited to children and women only as these two sub-sections of the population are most affected by anaemia [17, 18]. Very limited research is available examining anaemia among men in India and across various countries [19, 20]. Most of the studies examining anaemia among men in India are community-based [21, 22]. The limited literature on anaemia among men prompted us to include men in our study. Previously a few studies have examined the prevalence and etiology of anaemia among women, men, and children [19]. However, this study took a different approach and examined the triple burden of anaemia in the same household. To the best of our knowledge, this is the first study to examine the co-existence of the triple burden of anaemia among mother-father-child pairs in India.

## Methods

The study used a nationally representative fourth round of National Family Health Survey data, conducted in 2015–16 under the stewardship of the Ministry of Health and Family Welfare (MoHFW), Government of India. The survey provides information on population, health, and demographic aspects of households, men, women, and children for India as a whole, as well as for each state (29) and union territory (7) and district (640). A total of 601,509 households, 699,686 eligible women aged 15–49 years, and 112,122 men aged 15–54 years were interviewed with a response rate of 98%, 97%, and 92%, respectively. The detailed methodology, with complete information on the survey design and data collection, was published elsewhere [23]. The study excluded currently pregnant women’s sample from the analysis as the cut-off of anaemia for pregnant and non-pregnant women is different. The effective sample size for the study was 26,910 couples, along with children aged 6–59 months.

### Outcome variable

The outcome variable of the study was created with the combination of three dichotomous variables, namely, mother’s anaemia level (anaemic and not anaemic), father’s anaemia level (anaemic and not anaemic) and child’s anaemia level (anaemic and not anaemic). For the study purpose, a dichotomous variable was generated with the help of the above-mentioned variables. If the couple was anaemic along with the child, it was coded as one means “anaemic”, and if the couple was not anaemic along with the child, it was coded as 0 means “not anaemic”. Women’s blood haemoglobin level categorized as anaemic (< 12 g/dl) and not anaemic (≥12 g/dl), men’s blood haemoglobin level divided as anaemic (< 13 g/dl) and not anaemic (≥13 g/dl) [24]. Also, the study used children anaemia level, and categorized them as anaemic (< 11 g/dl) and not anaemic (≥11 g/dl) [25]. The outcome variable of the study was created with the combination of three dichotomous variables, namely, mother’s anaemia level (anaemic and not anaemic), father’s anaemia level (anaemic and not anaemic) and child’s anaemia level (anaemic and not anaemic) [23].

### Exposure variables

The study included couple’s age, couple’s education, couple’s employment status, caste, religion, wealth quintile, and regions as exposure variables. ‘Couple’s age was categorized as: both young (15–24 or < 25 years), both old (≥25 years), and others). Couple’s education was categorized as: both uneducated, both educated, and anyone educated. The working status of couples was grouped as: both unemployed, both working, and anyone working. Caste was divided into four categories: Scheduled Caste (SC), Scheduled Tribe (ST), Other Backward Caste (OBC), and others [26]. Religion was categorized as Hindu, Muslim, and others (including Christian, Sikh, Buddhist/Neo-Buddhist, Jain, Jewish, Parsi/Zoroastrian, no religion, and others). Wealth quintile was calculated by combining household’s amenities, assets, and durables and characteristics households in a range varying from lowest to the highest in a five-point Likert scale [27]. Place of residence was given as urban and rural. Geographical regions were categorized as North, Central, East, Northeast, West, and South [28].

### Statistical analysis

The bivariate and binary logistic regression analyses were applied to assess the factors associated with family-level anaemia. In bivariate analysis, a chi-square test was performed to determine the association of socio-demographic factors with anaemic family [29]. The adjusted odds ratio with a 95% confidence interval was presented in results.

The equation form of the model as follows:

$$ \ln \left(\frac{P_i}{1-{P}_i}\right)={\beta}_0+{\beta}_1{x}_1+\cdots +{\beta}_M{x}_{m-1} $$

Where *β*_0_, …. . , *β*_*M*_ are regression coefficient indicating the relative effect of a particular explanatory variable on the family anaemia. These coefficients change as per the context in the analysis in the study [30].

## Results

Table 1 represents the socio-demographic profile of the study population in India. It was found that 7% of couples were young, i.e., in the age group of less than 25 years. About 12.2% of couples were uneducated, whereas 20.5% of couples include anyone educated among them. Nearly 23.7% of couples belong to other caste categories, and about 80% of couples belong to the Hindu religion. Surprisingly, about 44% of a couple belongs from poor wealth quintiles, which include the poorest and poorer categories. Nearly 69% of couples belong to rural areas, and 25% of couples belong to central India.

**Table 1:** Socio-demographic profile of the study population, India, 2015–16

Background variables	Percentage	Sample
**Couple’s age**		
Both young	7.0	1849
Both old	67.4	19,014
Others	25.7	6047
**Couple’s education**		
Both uneducated	12.2	3517
Both educated	67.3	17,595
Anyone educated	20.5	5798
**Couple’s employment**		
Both unemployed	7.0	1949
Both employed	16.7	4618
Anyone employed	76.2	20,343
**Caste**		
Scheduled Caste	20.6	5034
Scheduled Tribe	11.0	5625
Other Backward Class	44.6	10,260
Others	23.7	5991
**Religion**		
Hindu	79.7	19,495
Muslim	15.5	4246
Others	4.7	3169
**Wealth quintile**		
Poor	43.8	12,735
Middle	21.2	5613
Rich	35.0	8562
**Place of residence**		
Urban	31.0	6954
Rural	69.0	19,956
**Region**		
North	13.4	5446
Central	25.1	7339
East	21.9	4843
Northeast	3.5	3747
West	15.7	2483
South	20.4	3052

**Fig. 1:**
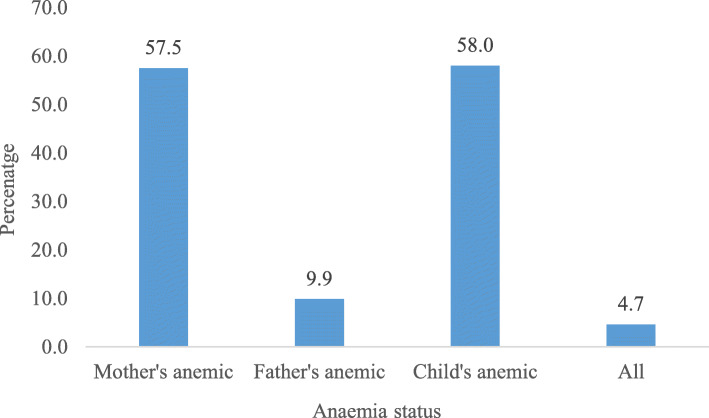
Percentage of anaemia among family members in India, 2015-16

Figure 1 reveals that almost 5 % of all family members were suffering from anaemia in India, which includes mother (57.5%), father (9.9%), and child (58%) as anaemic.

Table 2 reveals the percentage distribution of family-level anaemia (mother, father, and children) by background characteristics in India. The couple age difference was not found to be a significant factor for anaemia prevalence in a family. Nearly 7% of family anaemia was found if both mother and father were uneducated. Interestingly, anaemia prevalence was high (5.8%) in a family if both couples were employed. Anaemia prevalence was high in the family, which belongs to the Scheduled Tribe caste (8.3%). Families form the Hindu religion was having a higher prevalence of anaemia (4.8%). Families from Poor wealth status (6.4%) and rural areas (5.4%) were having a higher prevalence of anaemia. Families from the eastern part of India had the highest prevalence of anaemia (6.9%).

**Table 2:** Percent distribution of family-level anaemia (mother, father, and children) by background characteristics, India, 2015–16

Background variables	Anaemia (%)	Confidence Interval (CI)	***p***-value
**Couple’s age**			0.838
Both young	5.0	(4.07–6.07)	
Both old	4.5	(4.17–4.76)	
Others	5.1	(4.59–5.70)	
**Couple’s education**			0.001
Both uneducated	7.1	(6.28–7.98)	
Both educated	3.8	(3.58–4.15)	
Anyone educated	5.9	(5.27–6.48)	
**Couple’s employment**			0.002
Both unemployed	4.6	(3.76–5.63)	
Both employed	5.8	(5.10–6.45)	
Anyone employed	4.4	(4.16–4.72)	
**Caste**			0.001
Scheduled Caste	4.3	(3.77–4.89)	
Scheduled Tribe	8.3	(7.58–9.03)	
Other Backward Class	4.7	(4.23–5.04)	
Others	3.3	(2.90–3.81)	
**Religion**			0.094
Hindu	4.8	(4.56–5.17)	
Muslim	3.8	(3.23–4.38)	
Others	4.5	(3.74–5.18)	
**Wealth quintile**			0.001
Poor	6.4	(5.99–6.84)	
Middle	4.2	(3.75–4.81)	
Rich	2.7	(2.37–3.06)	
**Place of residence**			0.001
Urban	3.1	(2.69–3.50)	
Rural	5.4	(5.06–5.68)	
**Region**			0.001
North	4.1	(3.63–4.69)	
Central	4.9	(4.38–5.37)	
East	6.9	(6.24–7.67)	
Northeast	4.2	(3.55–4.84)	
West	3.1	(2.50–3.88)	
South	3.6	(2.87–4.18)	

%: Percentage; *CI* Confidence interval

Table 3 presents the logistic regression estimates for anaemic families by background characteristics in India. It was found that if couples, i.e., mother and father, both were educated, then the likelihood of anaemia in a family was low [OR: 0.69, CI: 0.58–0.81]. Additionally, if the mother or father, i.e., anyone among them, was educated, then the likelihood of anaemia in a family was low [OR: 0.82, CI: 0.0.70–0.97]. If both mother and father were employed, then the odds of anaemia in the family was high [OR: 1.40 CI: 1.10–1.80]. Families from the Scheduled Tribe had a 62% higher likelihood to suffer from anaemia [OR: 1.62, CI: 1.33–1.97]. Families from the rich wealth quintile were 44% less likely to suffer from anaemia than families from the poor wealth quintile [OR: 0.56, CI: 0.47–0.68]. Families from the eastern part of India were 9% more likely to suffer from anaemia than families in the northern part of India; however, the results were not significant [OR: 1.09, CI: 0.91–1.30]. Moreover, families from north-east India had a lower likelihood to suffer from anaemia than families from north India [OR: 0.36, CI: 0.28–0.47]. Moreover, the similar findings were found for other regions including central region [OR: 0.86, CI: 0.73–1.02], Western region [OR: 0.53, CI: 0.41–0.69] and Southern region [OR: 0.72, CI: 0.57–0.91].

**Table 3:** Estimates from binary logistic regression analysis for the anaemic family by background characteristics, India, 2015–16

Background variables	OR [95% CI]
**Couple’s age**	
Both young	Ref.
Both old	1.00(0.80–1.25)
Others	1.12(0.88–1.42)
**Couple’s education**	
Both uneducated	Ref.
Both educated	0.69***(0.58–0.81)
Anyone educated	0.82**(0.70–0.97)
**Couple’s employment**	
Both unemployed	Ref.
Both employed	1.40***(1.10–1.80)
Anyone employed	1.20(0.96–1.50)
**Caste**	
Scheduled Caste	0.92(0.75–1.13)
Scheduled Tribe	1.62***(1.33–1.97)
Other Backward Class	1.09(0.92–1.30)
Others	Ref.
**Religion**	
Hindu	Ref.
Muslim	0.93(0.78–1.10)
Others	1.17(0.93–1.48)
**Wealth quintile**	
Poor	Ref.
Middle	0.83**(0.71–0.97)
Rich	0.56***(0.47–0.68)
**Place of residence**	
Urban	Ref.
Rural	1.05(0.90–1.23)
**Region**	
North	Ref.
Central	0.86*(0.73–1.02)
East	1.09(0.91–1.30)
Northeast	0.36***(0.28–0.47)
West	0.53***(0.41–0.69)
South	0.72***(0.57–0.91)

*Ref*: Reference; *OR*: Odds ratio; *CI*: Confidence interval; ***p < 0.001; **p < 0.05; *p < 0.10

## Discussion

The main objective of the study was to examine the triple burden of anaemia in the same household where the focus was on the mother, father, and their last-born child. Result found that around 4.7% of households suffer from the triple burden of anaemia. Previously, various studies have examined the anaemia in mother-child pairs [31]; however, there is a scarcity of literature examining anaemia in mother-father-child pairs in the same household. We have examined the co-existence of anaemia among mother-father-child pairs at the household level. The study found that more than half of the mothers (57.5%) and children (58%) were anaemic. In contrast, only 10 % of fathers were anaemic. The prevalence of anaemia among mothers and children, as found in this study, is higher than the prevalence of anaemia in mothers and children in Nepal and lower than in Pakistan [32]. The prevalence of anaemia among children in Bangladesh was lower than in India, as found in this study [33]. This study illustrated that around 5 % of the mother-father-child pairs were anaemic in the same household. The co-existence of anaemia among mother-father-child pairs was found to be lower when both the parents were educated and the household belonged to rich wealth quintiles. In contrast, the co-existence of anaemia among mother-father-child pairs was higher when both the parents were employed and the household belonged to Scheduled Tribe.

This study has confirmed that with an increase in couple’s education, the triple burden of anaemia among mother-father-child pair’s decreases significantly. Previously, researchers have unanimously agreed in concluding that increasing the level of education has a negative association with anaemia [9, 34]. Child’s anaemia levels are widely affected by the mother’s level of education [9, 35]. Maternal education has found to be associated with an increased knowledge of healthcare and nutrition, which may possibly be linked to a reduction in anaemia level among members residing in a family [35]. Moreover, for child feeding practices, the mother’s education level is believed to be the key factor as mothers are the primary caregivers [36]. Further, it has been highlighted that likelihood of anaemia among children is higher when the anaemia levels are high among mothers [37, 38]. Therefore, it is understood that if a mother is anaemic, then the probability of a child being anaemic is high. Furthermore, severe anaemia among mothers may also impact the iron content in breast milk, which may lead to nutritional deficiency in the child and may aggravate anaemia among children [9]. A few studies have noticed a low level of anaemia among children with a higher father’s education level [39]; however, the pathways through which father’s education improves anaemia in their children are largely unexplored. One possible pathway suggests that educated parents may have a higher income, which may further have a bearing on improving a child’s nutrition and feeding practices [40].

Another important finding from this study highlighted that the triple burden of anaemia was higher when both the parents were employed as in comparison to when both the parents were unemployed. However, previous findings have concluded that the unemployment of either parent is positively associated with anaemia levels [41]. In relation to unemployment and anaemia, it can be understood that that unemployment among parents may lead to poor socio-economic status, which may worsen the nutritional intake and healthcare utilization and, as a result, aggravate the risk of anaemia [41]. Studies have noticed that employed parents have higher income, which betters the nutritional intakes and further has a bearing on the improvements of the anaemia level [42–44]. Assefa, Mossie, & Hamza (2014), in a community-based study, concluded that the problem of anaemia is linked with food insecurity, often characterized by economic constraints [43]. Moreover, some studies noticed no association between employment and anaemia [45]. However, in contrast to the previously available literature as discussed above, this study noticed a higher level of anaemia when both the parents were employed. The higher levels of anaemia when both the parents were employed may be attributed to the fact that when both the parents are working, they get less time in taking care of themselves along with their children and may fail in providing nutrition-rich foods. Employed parents find it tough to manage work-family conflict, and that may have an effect on child health, too [46].

Anaemia among mother-father-child pairs was higher among those who belonged to Scheduled Tribe. In the Indian context, Scheduled Tribes are socially and economically backward castes [47]. The tribal population was found to be at higher risk of under-nutrition because of their socio-economic conditions, and they also have poor health-seeking behavior [48]. Undernutrition among the tribal population is a well-known attribute of higher levels of anaemia among them [49]. Furthermore, the wealth quintile was found to be another predictor of anaemia among the study population. It was noticed that the triple burden of anaemia in a family decreases with an increase in their wealth. Previously, high levels of anaemia were found to be associated with the lowest wealth quintile [6, 50, 51]. Higher wealth in a household is directly linked to the higher nutritional purchasing parity [52]. The study concluded that the family-level anaemia burden was higher in the Northern region and lowered in the North-eastern, Western, and Southern regions of the country. A study has noticed a higher level of anaemia among children in the Northern region and lower levels of anaemia among children in Southern and North-eastern regions of India [52]. Jain & Agnihotri (2020), while examining nutritional outcomes in children, highlighted that Southern states like Tamil Nadu and Kerala were able to maintain balanced development in improving levels of anaemia over time by implementing programs like Integrated Child Development Scheme (ICDS) and other health missions in a holistic way [53].

The study has a few limitations. The cross-sectional nature of data limits our understanding to draw causal inferences from the findings, and hence, we could not follow the direction of causality. We could not examine district-level differentials as data for men was only available at the state level; hence, we examined regional differences only. The HemoCue device was used to test the capillary blood sample instead of venous blood in a laboratory; this may lead to an underestimation of anaemia [54]. In contrast, a study claimed that HemoCue provides an accurate measurement of anaemia [13]. While examining the co-existence of anaemia among mother-father-child pairs, we only measured anaemia for the last born child and not for all the children born to the couple. Furthermore, there may be a few unmeasured variables associated with anaemia we could not capture due to data limitation. Despite the above limitation, this study has several strengths. To the best of our knowledge, this is the first study that examined the triple burden of anaemia in a family by combining mother-father-child pairs in India. This study utilized nationally representative data, and hence, findings can be generalized at the national level.

## Conclusion

The study identified several risk factors that could be targeted to reduce the triple burden of anaemia among mother-father-child pairs in India. The limited availability of evidence in examining the triple burden of anaemia at the family level prompted us to suggest that there is a need to carry further studies in the related domain to conclude the findings evidently. The suggested interventions include early diagnosis, effective management, and treatment of anaemia. Moreover, adequate complementary feeding practices for children shall also be promoted. Parental education on nutrition is also required, and community interventions are needed to improve parental education on nutrition. Policy-makers may involve the local leaders or ASHA workers to disseminate nutrition education to the parents. At last, there is a need for greater policy and program attention to improving nutritional knowledge among mothers so as to tackle the triple burden of anaemia among mother-father-child pairs.

## Data Availability

The study utilises secondary source of data which is available on request and is available in public domain through. (https://dhsprogram.com/data/dataset/India_Standard-DHS_2015.cfm?flag=0)

## Abbreviations

NFHS: National Family Health Survey
OR: Odds Ratio
CI: Confidence Interval
ST: Scheduled Tribe
SC: Scheduled Caste
OBC: Other Backward Class
